# Water Vapor and Alcohol Vapor Induced Healing of the Nanostructured KBr Surface

**DOI:** 10.1021/acs.jpcc.2c03367

**Published:** 2022-07-27

**Authors:** Santanu Parida, Jesús S. Lacasa, Baran Eren

**Affiliations:** Department of Chemical and Biological Physics, Weizmann Institute of Science, 234 Herzl Street, 76100 Rehovot, Israel

## Abstract

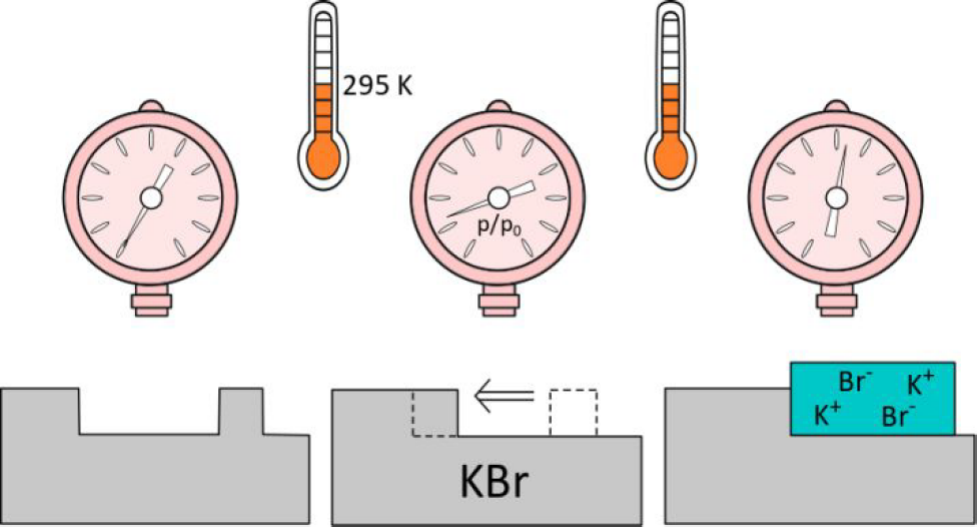

Using atomic force microscopy in the pressure range of 10^–10^ mbar to several tens of mbar at room temperature, we demonstrate the restructuring of nanostructured KBr surfaces assisted by the presence of water, methanol, and ethanol vapors and the formation of solvation islands. On a flat KBr surface, the two-dimensional solvation islands start nucleating at the step edges and grow with time and with increasing relative pressure. Solvation islands of water wet the terraces; however, solvation islands of methanol and ethanol are localized around the step edges and do not wet the terraces. Two processes are observed on nanostructured KBr surfaces: the movement of the atomic steps and the formation of solvation islands. The first process takes place at comparatively lower pressures at around 1% relative pressure, whereas the second process starts at higher pressures at around 25% relative pressure and above. Furthermore, the second process takes place only after the complete relocation of the step edges and thereby formation of a nearly flat surface. This implies that there is a competition between the restructuring of the atomic steps and solvation layer formation, as both processes require solvated ions. Unlike in the case of a flat surface, solvation islands of alcohols wet the restructured surface due to a higher density of low-coordination sites.

## 1 Introduction

Restructuring of surfaces in the presence of gases in the mbar pressure range is an interesting equilibrium property of solid surfaces that is so far mostly studied on single crystals of transition metals and thin oxide films supported on metals. For such atomically flat surfaces, high-pressure scanning tunneling microscopy (HP-STM) has been the main technique of choice.^1^ These studies have shown that at controlled ambient pressures and at room temperature (RT) and above even species with weak binding energy can have sufficient residence time on a surface that allows them to trigger reconstructions of the atomic structure; that is, under ambient conditions the surface structure dynamically adapts to its environment, and as a result completely new structures are often formed. In fact, even the most compact surfaces of some metals, which have the lowest energy configuration in a vacuum, were found to break up into clusters in the presence of gases at RT.^2^

The most obvious drawback of HP-STM is that it cannot be used to probe surfaces of electrically insulating materials. Unlike STM, atomic force microscopy (AFM) is not limited to electrically conductive materials. Experiments at controlled gas pressures ranging from ultrahigh vacuum (UHV) to 1 bar have been so far conducted with contact-mode AFM to study the frictional and tribological properties of carbon materials.^3,4^ Here, we expand the use of HP-AFM to non-contact-mode imaging.

Detailed studies on the restructuring of the surfaces of a large body of materials such as ionic crystals, wide band gap oxides, and so on are currently lacking. Alkali halide ionic crystals are an important group of insulating materials used in optics. Alkali halide crystals can also be used in future molecular electronic devices as substrate material because they allow direct access to the intrinsic electronic properties of the adsorbed molecules.^5^ Alkali halide surfaces have been nanostructured by various radiation treatments (e.g., e-beam, ion beam, plasma, etc.) to increase the density of undercoordinated step sites, where the preferential adsorption of molecules takes place, in a controlled manner.^5−10^ A major obstacle to utilizing alkali halides in molecular electronics is their stability in moist air because these surfaces have a high aqueous solubility and therefore could dissolve or even deliquesce in the presence of water vapor present in air above a certain relative pressure (deliquescence is the process of a solid fully dissolving due to the solvating influence of condensed water vapor). Relative pressure is defined as *p*/*p*_0_, where *p* is the absolute pressure and *p*_0_ is the vapor pressure of the gas, typically at RT. For water vapor, *p*/*p*_0_ is often termed relative humidity (RH).

The dissolution of alkali halides in air is an intriguing phenomenon observed in daily life with table salt as well as in many experiments with controlled RH. Already over half a century ago, it was discovered that cleaved surfaces of alkali halides have an RH-dependent superficial electrical conductivity, which was used to divide the water vapor–surface interactions into three domains.^11^ The first domain consists of physisorbed water with a submonolayer coverage on the surface, whereas solvation of ions takes place in the second domain, and the third domain is complete dissolution.^11^ After the advent of AFM, topography images of NaCl, KBr, and other alkali halide surfaces were obtained in controlled RH conditions.^12−18^ Some of these studies reported the movement of the steps on NaCl to start at around 40–45% RH at RT and associated it with the formation of the liquid solvation layer (also called hydration layer for water) on the surface, in agreement with the transition from the first domain to the second domain found in ref (11) and with infrared spectroscopy measurements in ref (19). The surface properties such as contact potential and friction coefficient change upon the formation of this liquid layer.^16,17^ On a more recent study, it was claimed that no dissolution of ions takes place below 30% RH.^20^ For KBr, this transition was reported around 55% HR,^11^ and both the moving steps and the formation of the hydration layer were observed above this threshold.^16,21^ In another recent study, large defects created by poking the KBr surface with an AFM tip were investigated.^22^ Interestingly, it was found that step movement already happens in the 3.0–5.5% HR range with a very sluggish kinetics and in the 12–20% HR range still with slow but more appreciable kinetics. This enhanced material transport was attributed to the material around the defect area no longer being in a monocrystalline configuration like the bulk material, thereby making it less stable and more mobile.^22^ We should mention that all the studies in the literature so far were conducted in air with controlled humidity, which contains considerable amounts of CO_2_ and trace gases like carboxylic acids that could affect the surface chemistry. Potential effects of such trace gases remain to be investigated.

In this study, we prepared a nanostructured KBr surface in UHV with a high density of monatomic steps using Ar ion sputtering and postannealing. In the presence of pure water vapor, we observed the movement of the steps already at the lowest pressure used in this work, *p*/*p*_0_ = 0.0043 (0.1 mbar at RT or 0.43% HR, RT is taken as 20 °C), with an increasing rate as the pressure is further increased. Such changes with discernible rates (in the order of few nanometers per hour) take place at higher relative pressures for methanol and ethanol; for instance, at *p*/*p*_0_ = 0.078 (10 mbar) for methanol and at *p*/*p*_0_ = 0.017 (1 mbar) for ethanol. We attribute this order of magnitude difference in partial pressure to higher polarity of water molecules which dissolves and removes the ions from the step edges more efficiently. These results imply that a nanostructured KBr surface is not stable in air even in relatively “dry” ambient conditions, and it is hard to realize them as templates for molecular electronics.

We also observe nucleation of two-dimensional solvation islands with all three gases. We believe that there is a competition between surface restructuring and formation of solvation layers, both of which use dissolved ions formed at the step edges. Our results show that surface restructuring is preferred over the nucleation of solvation islands; the latter starts only once the restructured surface becomes relatively flat in comparison to the nanostructured surface.

## 2 Experimental Section

All experiments were performed in UHV (base pressure between 1 × 10^–10^ and 5 × 10^–10^ mbar) conditions inside two chambers that are dedicated to sample preparation and AFM measurements. The preparation chamber is separated from the AFM chamber with a gate valve and is used for sputtering and annealing the samples and cantilever tips. An important feature of this system is the ability to switch the conditions in the AFM chamber from UHV to controlled ambient gas pressures, in either static or flow modes. Water (Milli-Q), methanol (spectrophotometric grade, ≥99.9%), and ethanol (absolute, ≥99.5%) vapors were dosed to the AFM chamber through a leak valve from a liquid reservoir, which was cleaned by several freeze–pump–thaw cycles before each experiment. AFM imaging was performed at static gas pressures, indicated as relative pressure in each image. We take *p*_0_ as 23.3, 129, and 58 mbar for water, methanol, and ethanol vapors, respectively.

Polished KBr(001) samples (from MaTeck) were cleaved in air prior to introducing them into vacuum chambers. They were then annealed at 250 °C (at the bottom of the sample) for 60 min to achieve a flat surface and desorb any contaminants. For nanostructuring their surfaces, they were exposed to an Ar^+^ beam with 1 keV energy for 3 min at normal incidence, followed by 60 min annealing at 200 °C. A fresh KBr sample was prepared for each set of experiments. The initial morphology of the nanostructured sample is a bit different in each set because the exact position of the ∼1 × 1 mm^2^ sample with respect to the Ar^+^ beam is slightly different in each case, and the thickness of the KBr crystal is a bit different in each case (affects actual surface temperature during annealing).

All AFM measurements were performed by using a custom-built AFM operating under UHV and controlled gas pressures at RT. Our AFM is similar to the design in ref (23), however with a regular piezo-tube scanner instead of a closed-loop scanner. Commercially available Si cantilevers (Budget Sensors Tap150Al-G, *k*_c_ ∼ 5 N m^–1^) were used as force sensors, which were annealed at 150 °C for 30 min in UHV prior to measurements. A second flexural (*f*_2_) resonance mode was used to obtain the surface topography at small oscillation amplitudes.^24^ Images were recorded both in frequency modulation (FM) and in amplitude modulation (AM); the latter mode is usually necessary for stable imaging at ambient conditions. AFM was controlled by Nanonis electronics. During the FM mode, the input signal is demodulated to amplitude and phase which are both kept constant by using a digital phased-locked loop (PLL) controller, and the frequency shift (Δ*f*_2_) is used by the feedback loop of the *z* controller. During the AM mode, the PLL was set to keep only the phase constant, and amplitude was used by the feedback loop of the *z* controller. In this mode, any contrast related to the changing interaction between the scanning tip and the surface is observed in the Δ*f*_2_ channel. Imaging parameters such as the free oscillation amplitude (*A*_0_) and set-point amplitude (*A*_2_) for the AM mode and *A*_2_ and set-point Δ*f*_2_ for the FM mode are indicated on each image. All measurements were performed at RT, which is around 23 °C inside our microscope chamber. The quality factor (*q* factor) of our cantilevers is around 6000 in UHV, which drops in the presence of gases due to damping, for example, to ∼4600 in 1 mbar of gas, ∼2600 in 10 mbar of gas, ∼2000 in 40 mbar of gas, and ∼500 in ambient air. Exact values depend on the cantilever and the type of gas that is used. A decreasing *q* factor requires increasing the excitation amplitude (either manually during AM imaging or via the feedback loop during FM imaging) to adjust *A*_0_ at each condition. Changing the *q* factor has no effect on *xy* calibration or *z* calibration because they depend on the piezoelectric response of the tube scanner (i.e., not related to cantilever mechanics). At each condition, frequency is swept prior to measurement to readjust the slight change in *f*_2_ due to the presence of gases.

## 3 Results and Discussion

### 3.1 Cleaved Surfaces

We start our analysis with reference measurements on flat KBr(001) surfaces.

**Figure 1:**
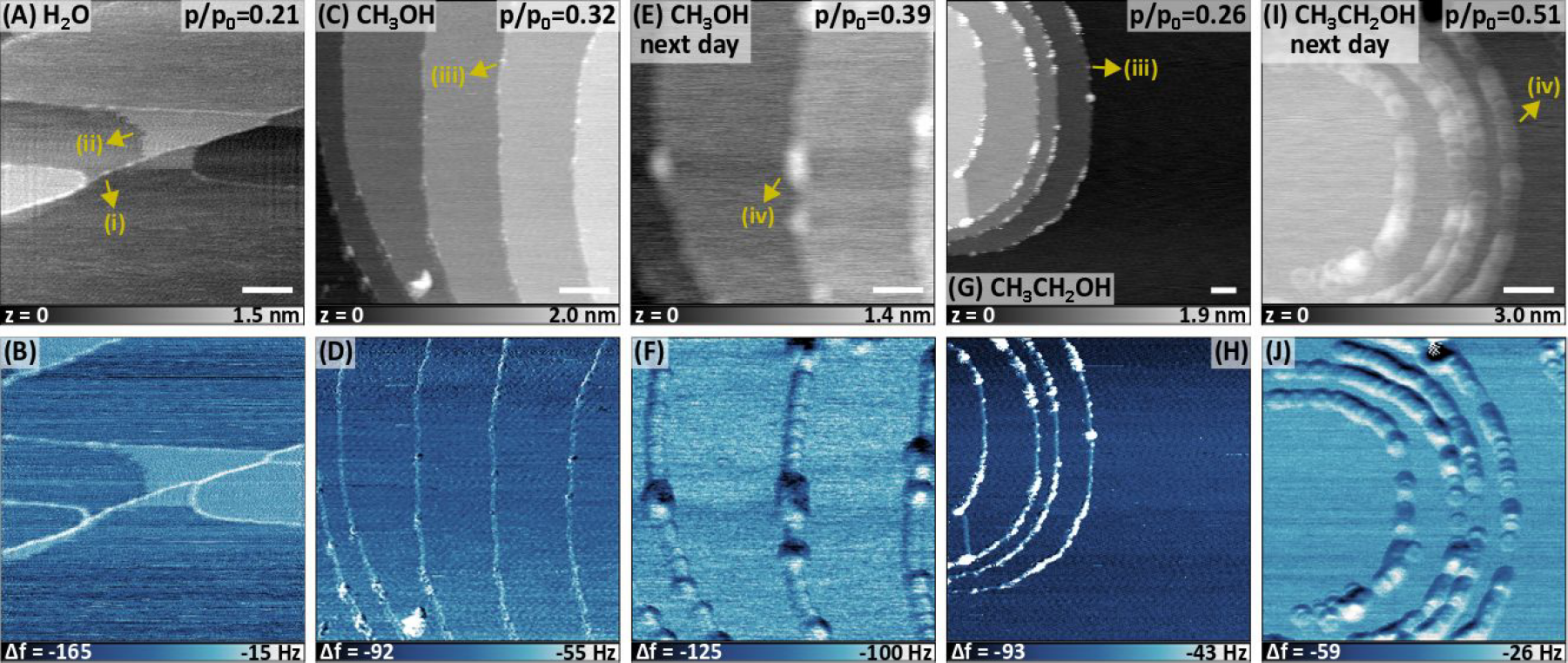
AFM images of the as-prepared KBr(001) surfaces exposed to different relative pressures of gas-phase solvents as indicated on image. The top row shows the topography, and the bottom row shows the Δ*f*_2_ channels. The scale bar is 100 nm in each image. (i) indicates a KBr step edge, whereas (ii) is the frizzled edge of the solvation layer. (iii) and (iv) show the solvation islands localized around the step edges for alcohol solvents. Because of the presence of gases, all images here were acquired in the AM mode with the following imaging parameters: *f*_2_ ≈ 0.78 MHz, *A*_0_ = 2.33 nm, *A*_2_ = 1.8 nm for (A); *f*_2_ ≈ 1.08 MHz, *A*_0_ = 3.4 nm, *A*_2_ = 2.7 nm for (C); *f*_2_ = 1.08 MHz, *A*_0_ = 3.6 nm, *A*_2_ = 2.7 nm for (E); *f*_2_ ≈ 0.935 MHz, *A*_0_ = 3.7 nm, *A*_2_ = 2.8 nm for (G); *f*_2_ ≈ 0.935 MHz, *A*_0_ = 4.2 nm, *A*_2_ = 3.0 nm for (I).

*Water:*Figures 1a and 1b show the topography and Δ*f* (Δ*f*_2_) images in the presence of water vapor at *p*/*p*_0_ = 0.21. The Δ*f* image exhibits some regions with a higher value (lower shift) that are due to the formation of solvation islands, as such regions are not observed in images obtained in UHV or at very low partial pressures. In fact, we observe the formation of solvation islands at as low as *p*/*p*_0_ = 0.043 (Figure S1), which is an order of magnitude lower than those reported in previous studies performed at ambient air conditions.^11,16,21^ Ambient air has a very poorly defined conditions that involve a large number of variable parameters;^21^ that is, it mainly consists of gases that should not have any significant interaction with nonpolar KBr surfaces, but there are also trace gases in air which might have appreciable interaction with the polar step edges of the KBr surface. For instance, carboxylic acids in air were found to be the reason behind ordered structures on the TiO_2_(110) surface,^25^ although such structures were initially attributed to water and water-related species.^26^ The effect of various other gases (e.g., CO_2_, carboxylic acids, etc.) on the nucleation of solvation layers needs further investigation. Our test experiments with water vapor that is kept in air for a few days show that a higher relative pressure is required for the nucleation of solvation layers on KBr(001) in such conditions. The thickness of the solvation islands in Figure 1a is around 0.25 nm, which is shorter than the 0.33 nm step height of the KBr(001) surface and suggests that they are single layer thick. We also observe an increase in the density of the areas covered with solvation islands with higher pressures and with longer exposure (e.g., overnight exposure). Moreover, in some areas, the height of the solvation islands become roughly double, indicative of the formation of a second layer. Once the chamber is evacuated from water vapor, we observe a rougher surface due to the precipitation of the K^+^ and Br^–^ ions of the solvation islands on the surface (Figure S2).

In our images, the solvation islands result in a brighter Δ*f* contrast in most cases (also for methanol and ethanol, as shown in examples later), which means a lower attractive interaction between the tip and the surface. Only in a very few images, we observed an opposite, darker contrast on the solvation islands that is indicative of stronger attractive interaction (Figure S1b). Because the tip could also be covered with water layers, its conditions are undefined. The mechanism leading to a brighter Δ*f* contrast for the solvation islands is probably not straightforward and eludes us at present.

*Alcohols:*Figures 1c and 1d show the topography and Δ*f* images in the presence of methanol vapor at *p*/*p*_0_ = 0.32, and Figures 1g and 1h show the same for ethanol vapor at *p*/*p*_0_ = 0.26. Both images show similar results, where the formation of solvation islands is localized around the step edges and do not nucleate further toward the terraces like they do for water. Only after increasing the pressure further and waiting overnight were the solvation islands found to increase in size (Figure 1e,f,i,j), but instead of wetting the surface they form 0.5–1.5 nm multilayer structures that are still localized around the step edges. They are also not well resolved because the tip gets covered with multilayers of alcohol molecules at such high pressures. While in initial phases the localized solvation islands result in a brighter Δ*f* contrast similar to the solvation islands of water (Figure 1d,h), the height of the structures gets convoluted into the Δ*f* signal in later stages (Figure 1f,j).

In brief, reference images in Figure 1 show that solvation islands nucleate and wet the flat KBr(001) surface, but at a lower relative pressure for pure water vapor than those reported in the literature for humid air. Methanol and ethanol vapors also form solvation islands on KBr(001) but are highly localized around the step edges. Instead of wetting and thereby covering the surface uniformly, they form multilayer structures.

### 3.2 Water Vapor

**Figure 2:**
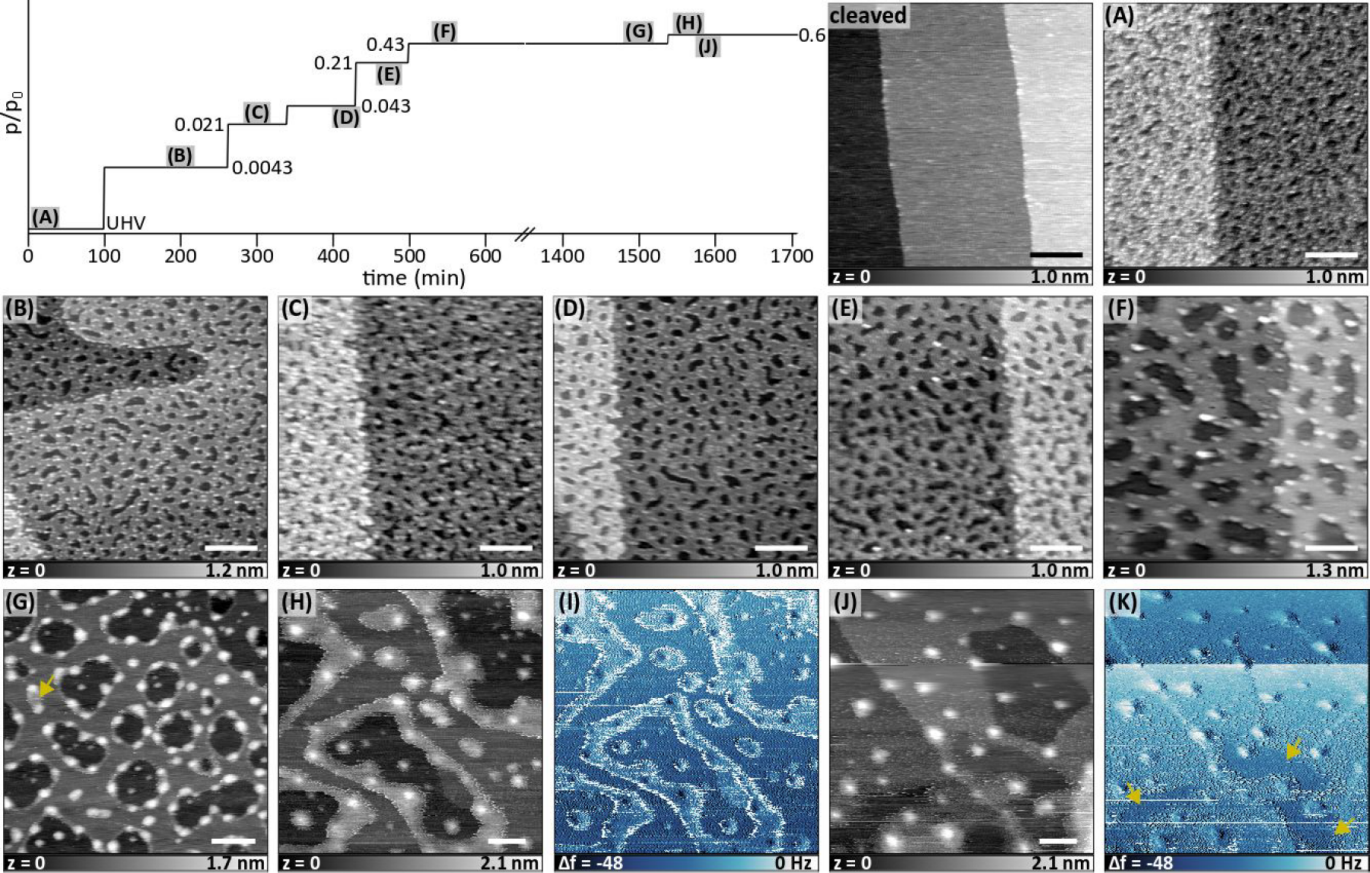
AFM images of the nanostructured KBr surface as a function of the relative pressure of water vapor, which are indicated in the *p*/*p*_0_ vs time graph at the top left. Actual pressures are normalized to 23.3 mbar to estimate *p*/*p*_0_ (i.e., RH). Prior to dosing water, first two measurements were performed in UHV, initially on the cleaved sample and then on the nanostructured sample (A). (B–H) and (J) are topography images, whereas (I) and (K) are the Δ*f* images corresponding to (H) and (J), respectively. The scale bar is 100 nm in each image. Imaging parameters: *f*_2_ ≈ 0.792 MHz; (A) is in FM mode, Δ*f*_2_ = −50 Hz, *A*_2_ = 3.5 nm; the rest are in AM mode with *A*_0_ = 3.5 nm, *A*_2_ = 3.1 nm for the cleaved sample, *A*_0_ = 3.5 nm, *A*_2_ = 3.2 nm for (B), *A*_0_ = 3.5 nm, *A*_2_ = 3.15 nm for (C), *A*_0_ = 3.5 nm, *A*_2_ = 3.25 nm for (D), *A*_0_ = 3.85 nm, *A*_2_ = 3.2 nm for (E), *A*_0_ = 3.55 nm, *A*_2_ = 2.6 nm for (F), *A*_0_ = 2.9 nm, *A*_2_ = 2.45 nm for (G), *A*_0_ = 2.4 nm, *A*_2_ = 1.9 nm for (H), and *A*_0_ = 2.4 nm, *A*_2_ = 1.6 nm for (J). Solvation islands are predominantly at the step edges in (G), except for the small region indicated with an arrow. In (H), solvation islands start covering the terraces near the step edges, which results in a brighter Δ*f* in (I). In (J), most of the surface is covered with the solvation layer, except for the darker contrast Δ*f* regions indicated with arrows in (K). The tip is less stable at higher pressures due to damping and capillary bridges formed between the tip and the sample; for instance, there is a change in the tip during imaging of (J) and (K) which changes Δ*f* abruptly.

We start our main discussion on the nanostructured KBr surfaces with water vapor, as it is the most relevant gas for optics and molecular electronics applications. Figure 2a shows the topography image of the nanostructured KBr surface in UHV (image of the same surface prior to nanostructuring is shown on the left of Figure 2a), and Figure 2b–f shows similar images in the presence of water vapor with the relative pressures ranging from *p*/*p*_0_ = 0.0043 to 0.43 as indicated in the *p*/*p*_0_ versus time graph in Figure 2. As-prepared surface in UHV consists of pits with monatomic thickness (Figure 2a). These pits are different from those formed via electron deposition that have step edges oriented along the nonpolar ⟨100⟩ directions.^7^ The step edges of the pits in Figure 2a are random in shape and orientation and thus have some polar character. While the nanostructured surface is stable in UHV for days, pressure-dependent differences in topography are apparent in AFM images (Figure 2b–f): the small pits with monatomic thickness merge into one another, leading to the surface becoming less corrugated with increasing pressure. In addition to the pits, there are also poorly resolved, small, round protrusions on the surface that have increasing lateral size with increasing pressure. The height of some of these protrusions are less than that of a KBr(001) step, which might suggest that they are a different type of material than KBr. Unfortunately, the topography is convoluted into the Δ*f* signal, and therefore it is difficult to use Δ*f* for material contrast for such protrusions.

It is remarkable that the surface structure of the nanostructured KBr(001) changes already at very low relative pressures. It suggests that already at *p*/*p*_0_ = 0.0043 (0.1 mbar) either K^+^, Br^–^, or both ions are dissolved by the physisorbed water molecules. These ions are mobile on the surface, until they reach a step edge and coalesce to that step edge. A gradual self-repair mechanism of the KBr(001) surface upon poking it with an AFM tip was reported in ambient conditions at much higher partial pressures of water (*p*/*p*_0_ = 0.12–20) in ref (22). Our results show that ion dissolution takes place even at lower pressures in the pressure regime that is traditionally attributed to “physisorbed water molecules”. In other words, destabilization of the ions at the steps and the nucleation of a solvation layer do not necessarily happen simultaneously. On flat KBr(001) surfaces, it is difficult to observe the motion of the step edges because the dissolved ions likely deposit back to their original position instead of repairing a more corrugated step edge. Moreover, the step edges of the flat KBr(001) surface are either nonpolar or less polar than those of the nanostructured surface. Because edges of the monatomic pits are unstable in the presence of water vapor, so should the round protrusions if they were made of KBr. Because of this and the previously mentioned reason about their height, we conjecture that these are in fact metallic K clusters, which nucleate from the mobile K^+^ ions on the surface. Unlike K, Br cannot form such clusters as it will form Br_2_ and desorb to the gas phase at RT. Previous X-ray photoelectron spectroscopy (XPS) studies indeed showed that the KBr surface becomes richer in K upon exposure to water vapor, but such studies are not conclusive because of the beam-induced effects on alkali halides.^27^

Another remarkable observation is the lack of two-dimensional solvation islands despite the relative pressure range being as high as 0.43, an order of magnitude higher than the partial pressure for solvation island formation on the flat KBr(001) surface (Figure S1). Figure 2g shows the topography image of the initially nanostructured surface left overnight at *p*/*p*_0_ = 0.43. As expected, the surface becomes even flatter (less frequent but larger pits) due to self-repair, and the round particles that we attribute to metallic K increase in size. We also start observing the solvation islands around the step edges and on a few places on the terraces (one of them is indicated with an arrow in Figure 2g). These results suggest that in the temperature–pressure conditions of this study both the formation of the solvation layer and self-repair are thermodynamically preferred over the nanostructured surface. However, they are competing processes as they both require availability of dissolved ions on the surface, and our results highlight that self-repair is preferred over the formation of solvation layers. This is why although the formation of solvation islands is not kinetically limited when the relative pressure is over *p*/*p*_0_ = 0.043 (as suggested by the measurements on flat KBr(001)), it does not take place until the surface reconstruction from a nanostructured surface into a flat surface is nearly completed. Figure 2h shows the topography image when the relative pressure is further increased to 0.6. A solvation layer with a thickness of ∼0.5 nm propagates from the step edges onto the terraces, which also results in a brighter Δ*f* contrast in Figure 2i. Once the solvation layer nucleates on the terraces, the entire surface eventually gets covered with this layer depending on the relative pressure (thereby the chemical potential of water vapor) and duration (slow kinetics at low pressures): Figures 2j and 2k show the surface after 20–30 min storage at *p*/*p*_0_ = 0.6, which is almost entirely covered with the solvation layer except for the regions shown with arrows in Figure 2k. The height difference caused by the solvation layer in Figure 2j is roughly between 0.25 and 0.5 nm, depending on the number of solvation layers available on the region. Images in Figure 2h–k are noisier than other images due to a lower *q* factor of the cantilever, mobility of the solvated ions and water molecules inside the solvation layer, and capillary forces between the tip and sample. This is also the case for images that are shown below with alcohols at higher pressure.

### 3.3 Vapors of Alcohols

**Figure 3:**
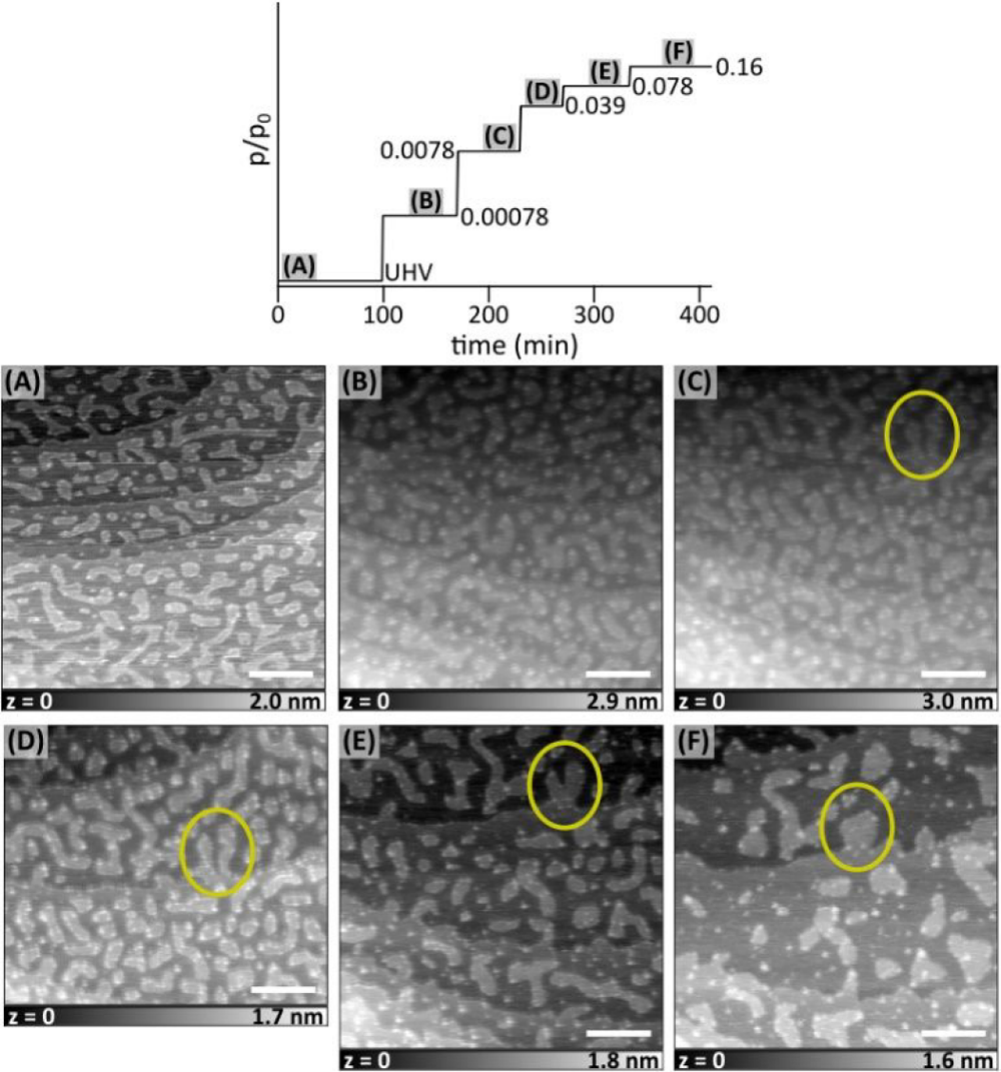
AFM topography images of the nanostructured KBr surface as a function of the relative pressure of methanol vapor, which are indicated in the *p*/*p*_0_ vs time graph at the top. Actual pressures are normalized to 129 mbar to estimate *p*/*p*_0_. The scale bar is 100 nm in each image. Yellow circles indicate the same position in different images, in which coalescence of two islands take place. Images on (A), (B), and (C–F) are on different regions. Imaging parameters: *f*_2_ ≈ 0.852 MHz; (B) and (D) are in FM mode, Δ*f*_2_ = −40 Hz, *A*_2_ = 4.0 nm; the rest are in AM mode with *A*_0_ = 4.6 nm, *A*_2_ = 4.0 nm for (A), *A*_0_ = 4.0 nm, *A*_2_ = 3.7 nm for (C), *A*_0_ = 4.75 nm, *A*_2_ = 4.2 nm for (E), *A*_0_ = 4.55 nm, *A*_2_ = 3.6 nm for (F). No solvation layer is apparent in any of the images.

Figure 3 shows the changes in the nanostructured KBr surface in the presence of methanol vapor at various relative pressures. The as-prepared sample has connected pits compared to that shown in Figure 2a, likely due to a difference in the exact position of the sample with respect to the Ar^+^ beam and because the actual temperature on the sample surface is different during annealing. Unlike in the case of water, we do not observe any appreciable movement of the step edges at low relative partial pressures up to *p*/*p*_0_ = 0.039 (Figure 3a–d). Around *p*/*p*_0_ = 0.078 the small islands on the surface are consumed at the expense of larger ones similar to the Ostwald ripening process (Figure 3e), which is even more prominent when the relative pressure is further increased to 0.16 (Figure 3f). The yellow circles in Figure 3d–f are a guide for the eye for the same location on the surface, where the coalescence of two peninsulas can be seen (also notice that there are no noticeable differences between Figures 3c and 3d). According to ref (28), the solubility (grams of salt per 100 g of saturated solution) of KBr is 40.7 for water, 2.06 for methanol, and 0.14 for ethanol. It is difficult to apply these values to our case because of two main reasons: The density of physisorbed water or alcohol layer on the surface is several orders of magnitude less than that of liquids, and geometrical constraints that are due to the two-dimensionality of our system. In other words, water or alcohol molecules can only attach to a K^+^ or Br^–^ ion at a step or kink sites either from the top or from the side. Nevertheless, the solubility values could be used as a first-order approximation to explain one order of difference in partial pressures needed to achieve a step edge motion for water versus for methanol.

**Figure 4:**
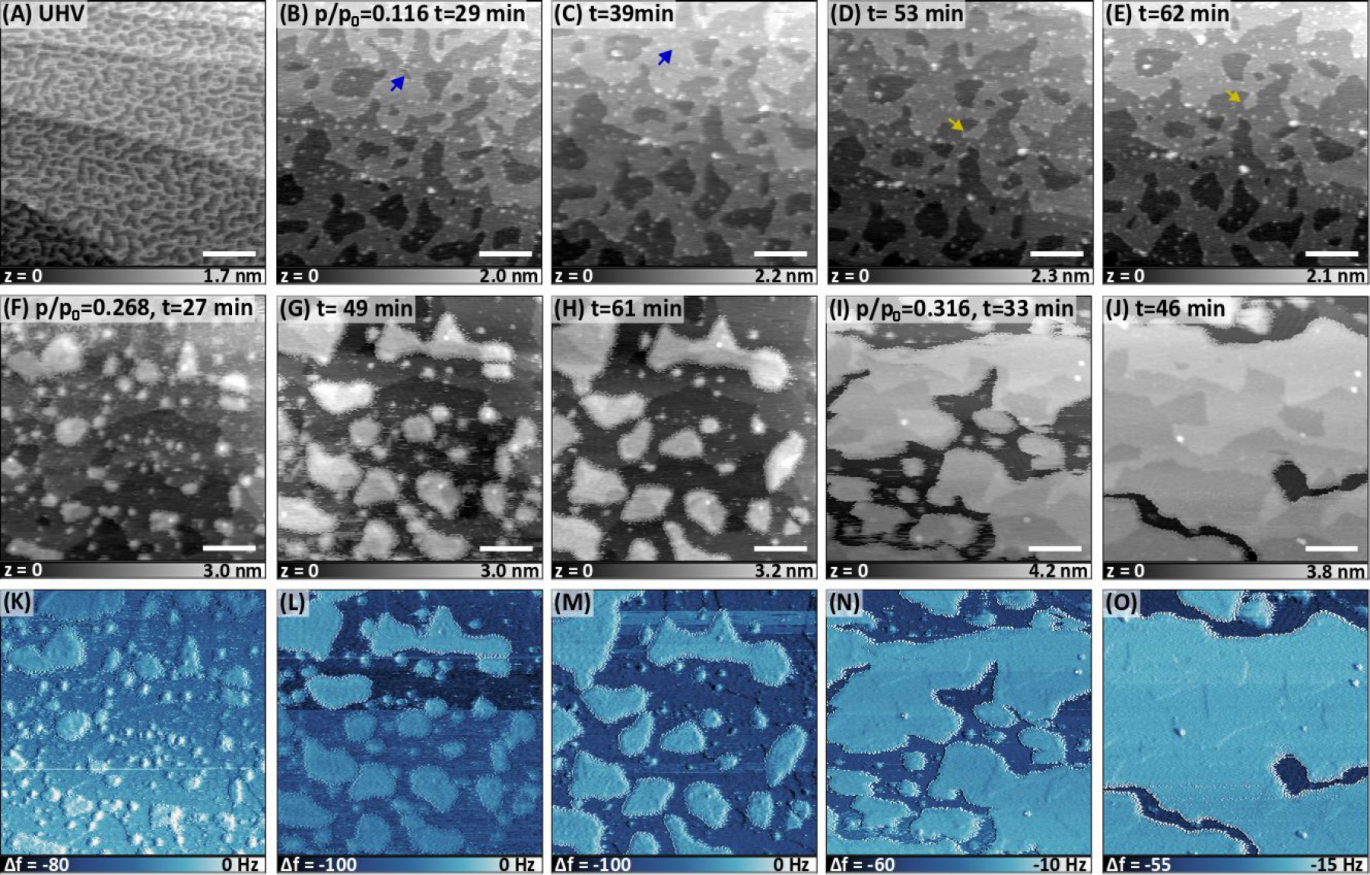
AFM images of the nanostructured KBr surface as a function of the relative pressure of methanol vapor. Actual pressures are normalized to 129 mbar to estimate *p*/*p*_0_, as indicated in each image. At *p*/*p*_0_ = 0.268 and higher, solvation layers form and start covering certain regions of the surface, which result in a brighter Δ*f* contrast than uncovered surfaces. (B) to (E), (F) to (H), and (I) to (J) are shown as time-lapse topography images to show time-dependent changes in surface morphology. The last row of images (K–O) show the Δ*f* images corresponding to the topography images in the middle row. In (B) to (E), arrows indicate some of the changes between time-lapse images. The scale bar is 100 nm in each image. Imaging parameters: *f*_2_ ≈ 0.935 MHz; (A) in FM mode with Δ*f*_2_ = −40 Hz, *A*_2_ = 4.0 nm; the rest are in AM mode with *A*_0_ = 4.85 nm, *A*_2_ = 4.0 nm for (B) and (C), *A*_0_ = 4.85 nm, *A*_2_ = 3.9 nm for (D), *A*_0_ = 4.85 nm, *A*_2_ = 3.8 nm for (E), *A*_0_ = 4.82 nm, *A*_2_ = 3.0 nm for (F), *A*_0_ = 4.82 nm, *A*_2_ = 2.6 nm for (G) and (H), and *A*_0_ = 4.81 nm, *A*_2_ = 3.7 nm for (I) and (J).

We investigated the effect of methanol vapor further at higher pressures on the nanostructured KBr surface using another sample that also consists of monatomic thick pits that are partially connected to each other (Figure 4a). Figure 4b–e shows the time-lapse topography images of this surface in the presence of methanol vapor with *p*/*p*_0_ = 0.116. Upon initial dosing of methanol vapor, the surface reconstructs in the form of merging pits and becomes flatter. Time-lapse images show that the steady state is not reached in ∼1 h: small pits are consumed at the expense of larger ones, and step edges are still mobile as indicated with arrows. Upon increasing the relative pressure to 0.268, solvation islands start to nucleate on the surface (Figures 4f), with a brighter contrast in the Δ*f* image in Figure 4k. Figures 4g,h and 4l–m show the growth of the solvation islands with time, whereas Figures 4i,j and 4n–o show the same at *p*/*p*_0_ = 0.316 as the solvation layer almost fully occupies the terraces in less than an hour. This is essentially the same behavior as in the case of water, where nucleation of the solvation islands only starts once the self-repair of the surface via step motion is nearly completed. The step density of the repaired surface is still larger than that of a cleaved surface, and therefore the surface is still rich in regions where nucleation of the solvation islands can take place. The solvation islands in Figure 4f have a thickness of around 1 nm, which resemble the localized solvated layers on the flat surface (Figure 1e) but some of them with a significantly larger diameter. Unlike on the flat surface, these islands coalesce with each other and the solvation layer eventually wets the surface. The thickness of the solvation layer that is wetting the surface in Figure 4i,j is around 1.5 nm.

**Figure 5:**
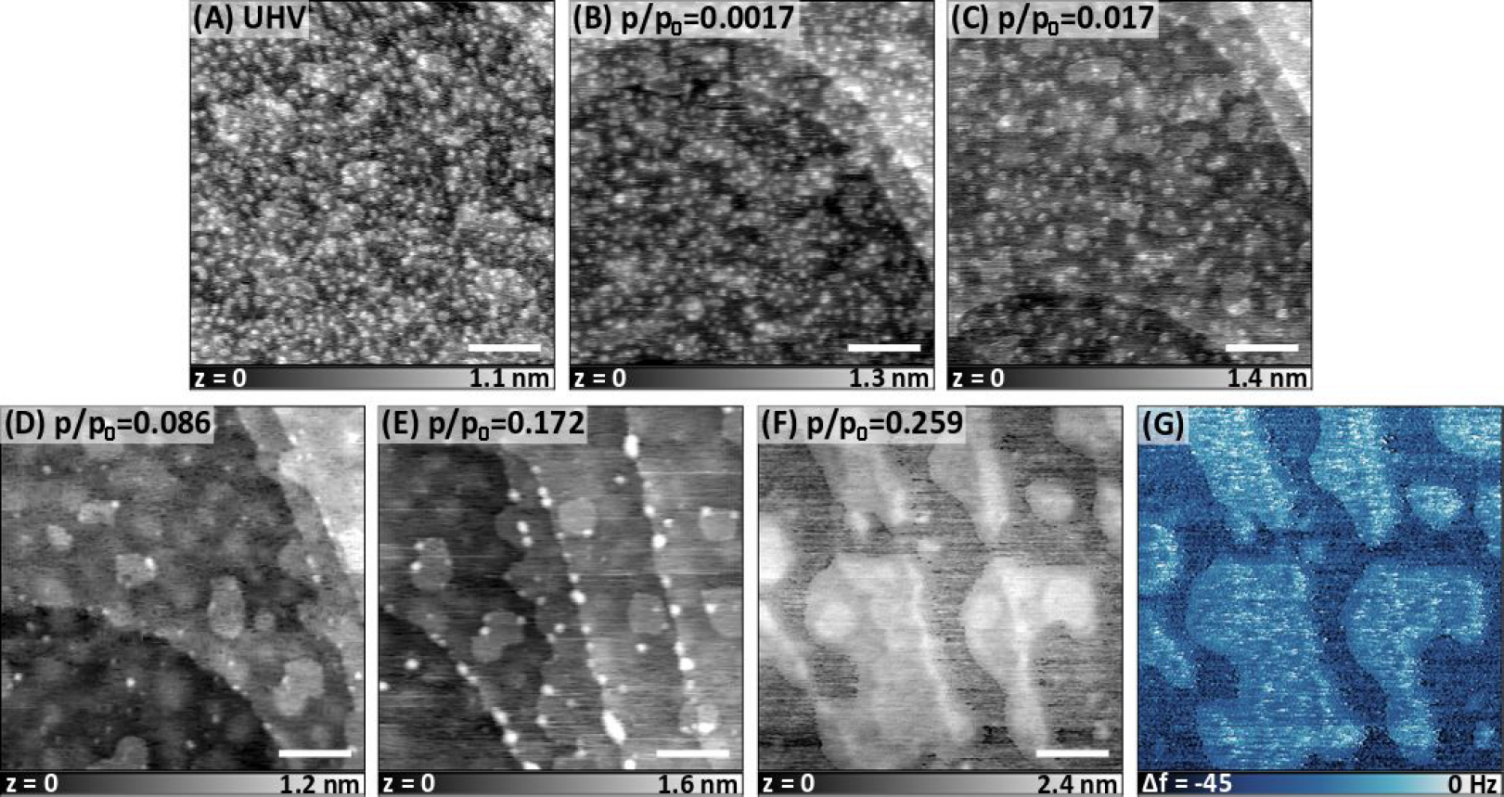
AFM images of the nanostructured KBr surface as a function of the relative pressure of ethanol vapor. Actual pressures are normalized to 58 mbar to estimate *p*/*p*_0_, as indicated in each image. Solvation layer formation was first observed at *p*/*p*_0_ = 0.259, which changes the Δ*f* contrast. Scale bar is 100 nm in each image. Imaging parameters: *f*_2_ ≈ 0.935 MHz; all images are in AM mode with *A*_0_ = 4.0 nm, *A*_2_ = 3.5 nm for (A) and (B), *A*_0_ = 4.15 nm, *A*_2_ = 3.3 nm for (C), *A*_0_ = 4.4 nm, *A*_2_ = 3.5 nm for (D), *A*_0_ = 4.3 nm, *A*_2_ = 3.5 nm for (E), and *A*_0_ = 4.55 nm, *A*_2_ = 3.5 nm for (F).

Lastly, we investigated the effect on ethanol vapor on the nanostructured KBr surface. Figure 5a shows the initial surface structure, which is slightly different from the previous structures. Similar to previous observations, there is no major change at low partial pressures (e.g., *p*/*p*_0_ = 0.0017 in Figure 5b), whereas gradual self-repair of the surface takes place for *p*/*p*_0_ = 0.017–0.172 with faster kinetics at higher pressure (Figure 5c–e). Formation of the solvation islands were observed on a relatively flat surface compared to the initial nanostructured surface (Figure 5f,g) at *p*/*p*_0_ = 0.259. As in the case of methanol, solvation islands could wet the surface which was not observed for a flat, cleaved surface (Figure 1g–j). We cannot explain the self-repair mechanism of the surface in the presence of ethanol by solely relying on the solubility in liquids, which is 1 order of magnitude lower than that of methanol. At low relative partial pressures, both alcohol molecules adsorb on the surface through the van der Waals interactions. Because ethanol is a larger molecule, van der Waals interactions with the surface should be higher for ethanol compared to methanol. Therefore, equilibrium coverage of ethanol should be higher than equilibrium coverage of methanol, increasing the possibility of ion dissolution at the step edges despite lower polarity.

## 4 Conclusions

Water, methanol, and ethanol vapors result in step motion on the nanostructured KBr surfaces due to the dissolution of the ions at the step edges. This is a self-repair mechanism that results in a flatter surface, but not as flat as a cleaved KBr(001) surface. This is a remarkable behavior as it shows that even physisorbed water and alcohol molecules are sufficient to initiate ion dissolution. Formation of solvation islands also requires the dissolved ions, but it takes place at higher pressures compared to the movement of the steps. Compared to nanostructured surfaces, alcohol molecules only form localized solvation islands around the step edges on a flat surface, and these island do not wet the surface.

This benchmark study of KBr(001) highlights that atomic rearrangements on alkali halide surfaces can take place even at 0.1 mbar water vapor pressure, equivalent to <1% RH. This behavior could render them impractical for molecular electronics applications even in dry air. Such atomic rearrangements were previously reported for metallic surfaces and thin oxide films. As more surface-sensitive microscopy, spectroscopy, and diffraction techniques becomes available for studies in controlled gas environments, we can learn more about the actual atomic, chemical, and electronic structure of surfaces in equilibrium with their surroundings.

## Supporting Information Available

Additional AFM images (PDF)
