# Supplementary material for: Water Vapor and Alcohol Vapor Induced Healing of the Nanostructured KBr Surface
Source: J Phys Chem C Nanomater Interfaces. 2022 Jul 27;126(31):13433–40. doi: 10.1021/acs.jpcc.2c03367 (PMC9377354; doi:10.1021/acs.jpcc.2c03367)
Supplement: Supplementary file 1 — jp2c03367_si_001.pdf [file jp2c03367_si_001.pdf]

Supporting Information for

'Water Vapor and Alcohol Vapor Induced Healing of the Nanostructured KBr Surface'

Santanu Parida, Jesús S. Lacasa, Baran Eren

Department of Chemical and Biological Physics, Weizmann Institute of Science, 234 Herzl Street, 76100 Rehovot, Israel,

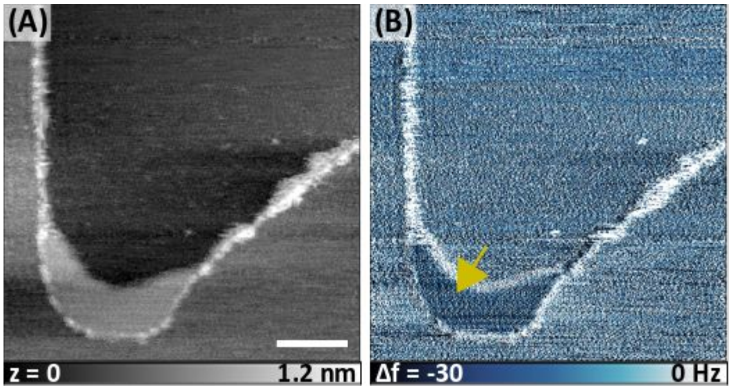

Figure S1 (a-b) AFM topography and Δf images of the KBr(001) surface in the presence of water vapor at p/p_0=0.043 (1 mbar) at RT. Solvation islands start forming near the step edges. Unlike in all the other images presented in the main text, the solvation islands have a darker Δf contrast in this image due to different tip conditions. Such a contrast is uncommon and was only seen in a few images among hundreds. Scale bar is 100 nm. Imaging was performed in AM mode.

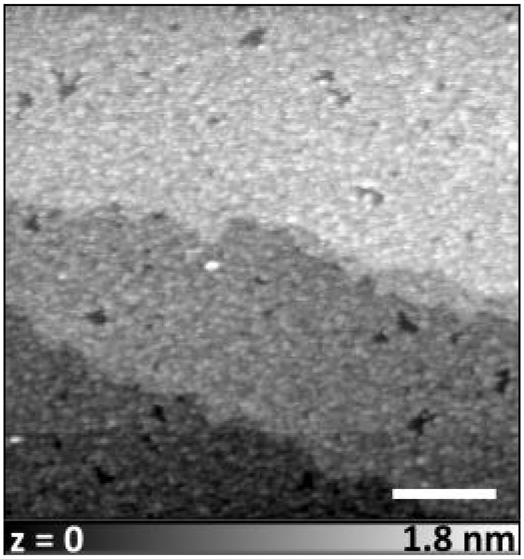

Figure S2 AFM topography image of the KBr surface after pumping out the water vapor from the chamber. Scale bar is 200 nm. Imaging was performed in FM mode.
